# Effect of modified indwelling needle insertion techniques in Chinese children: a systematic review and meta-analysis

**DOI:** 10.3389/fped.2025.1579303

**Published:** 2025-07-09

**Authors:** Hongmei Hou, Linhui Chen, Liping Liu, Xiangyu Yan, Huan Li, Xinyu Pan, Mengyan Jin

**Affiliations:** ^1^ Department of Neurology, The Third Affiliated Hospital of Chengdu Medical College, Chengdu Pidu District People’s Hospital, Chengdu, Sichuan, China; ^2^ Department of Evidence-Based Medicine and Social Medicine, School of Public Health Chengdu Medical College, Chengdu, Sichuan, China; ^3^ School of Medicine, University of Electronic Science and Technology of China, Chengdu, Sichuan, China; ^4^ Department of Pharmacy, Chengdu Medical College, Chengdu, Sichuan, China; ^5^ Department of Internal Medicine, West China Hospital, Sichuan University, Chengdu, Sichuan, China

**Keywords:** indwelling needle, puncture success rate, parents' satisfaction, randomized controlled studies, indwelling time

## Abstract

**Aim:**

A comprehensive study evaluated the effect of modified indwelling needle technique on puncture success rate and indwelling time in Chinese children.

**Background:**

Compared with ordinary steel needles, indwelling needles have many advantages. The incidence of complications of an indwelling needle is lower than that of an ordinary steel needle. There are many ways to improve it, such as a modified fixation and liquid connection indwelling needle, and many studies have conducted randomized controlled trials.

**Design:**

meta-analysis

**Methods:**

Databases including PubMed, Web of Science, CNKI, Wang Fang, CQVIP, Embase were electronically searched for studies on modified indwelling needle insertion techniques from inception to June 20, 2024. Two authors conducted the article search independently. A third author addressed the inconsistencies that arose between the two authors. Keywords were used for retrieval, and Boolean operators were used accordingly.

**Results:**

A total of 18 randomized controlled (RCT) studies were included. The results showed that the puncture success rate, indwelling time and parent satisfaction of the modified indwelling needle insertion technique were better than the control group. Sensitivity analysis was carried out after each study was excluded one by one, and the results showed that pooled sensitivity and specificity had no significant change, indicating that the stability of the meta-analysis, and no publication bias found.

**Conclusions:**

The modified indwelling needle technique showed increased puncture success rate, prolonged indwelling time and improved parental satisfaction compared with the control group.

## Introduction

The intravenous indwelling needle is the intravenous trocar needle. The core components include a soft sleeve that can be retained in the vessel, and a puncture guide needle core of stainless steel (1). In use, the catheter and the needle core are punctured into the blood vessel together. When all the catheter enters the blood vessel, the needle core is withdrawn, and only the soft catheter is retained in the blood vessel for infusion treatment (2).

Compared with ordinary steel needles, indwelling needles have many advantages. First, from a structural point of view, the indwelling needle has a reasonable structure, convenient operation (3), and a low leakage rate of infusion. The front end of the indwelling needle is a hose, which is convenient for the patient's limbs to move and is not easy to run, while the complication rate of the indwelling needle is less than that of an ordinary steel needle (4). To reduce the number of punctures, indwelling needles are commonly used in clinics or hospitals for blood collection and infusion therapy (5). Second, for patients, an indwelling needle can reduce the pain of multiple punctures, and it is widely used in paediatric wards (3). Reduce the pain caused by repeated venipuncture and the fear of injection, reduce the anxiety of parents, and enhance parents’ satisfaction (6). Meanwhile, for the medical staff, the use of indwelling needles enables them to quickly establish venous channels and improve the efficiency of saving a patient in an emergency (7), and, reduces the workload of nurse puncture, and liberates the time to have more energy for other rescue work (5).

But the indwelling needle is not all beneficial; for example, its cost is two to three times more than that of the ordinary steel needle. Indwelling needles that stay in the body for a long time will also have complications such as exudation, necrosis, phlebitis, catheter blockage, embolism, etc (8). Due to the special structure of the indwelling needle, there are high-level requirements for the nurse to be capable in puncture technology, and a successful puncture is required (9). However, it is worth noting that the core of the intravenous indwelling needle was not pulled out after the infusion, which resulted in children being unable to bathe normally and unable to carry out strenuous activities (10). At the same time, it may also be due to the violent action of patients, and the fixation of the indwelling needle is equally important (11).

The research on the modified indwelling needle focuses on the improvement of its core components, that is, the improvement of the catheter material, infusion joint and other own materials. Improving retention has a positive effect on patients and reduces complications. In addition, the success rate of puncture and indwelling time were improved by improving the indwelling needle technique. From each separate study, the modified technique had a positive effect on patients, which was better than the control group without the improved technique. However, the effects of the modified needle are different, and there is a lack of comprehensive analysis of the impact of the modified indwelling needle technique on patients.

Therefore, a meta-analysis, a systematic and comprehensive analysis, along with the evaluation of the modified indwelling needle technique were undertaken and the results combined to provide reference for clinical practice.

## Methods

### Search strategies

This study was a systematic review and meta-analysis conducted in accordance with the Preferred Reporting Items for Systematic Reviews and Meta-Analyses (PRISMA) statement (12). A systematic search of related studies was conducted across multiple electronic databases, including PubMed, Web of Science, China National Knowledge Infrastructure(CNKI), Wang Fang, China Science and Technology Journal Database(VIP), and Embase. The search encompassed studies published in English up to June 20, 2024. Two reviewers independently performed the literature search, and any discrepancies between them were resolved by a third reviewer. Keywords were employed for retrieval, and Boolean operators were utilized to refine the search strategy. The theme determination and search strategy follow the PICO principle, and the specific design is as follows: P: Children/Kids; I: Intravenous infusion adopts the modified indwelling needle technique; C: The control group mainly consisted of children who did not receive intravenous infusion with the modified indwelling needle. O: the puncture success rate, indwelling time and parent satisfaction of the modified indwelling needle insertion technique. The included research designs were mainly randomized controlled trials. The database retrieval strategies for other databases are detailed in Supplementary Table S2.

The PubMed search string used was:

1. (intravenous detaining needle [Title/Abstract]) OR (intravenous indwelling needle [Title/Abstract]) OR (venous indwelling needle [Title/Abstract]) OR (venous indwelling trocar [Title/Abstract]) OR (Indwelling needle [Title/Abstract]) OR (PVIN [Title/Abstract]) OR (peripheral venous catheter [MeSH Terms])
2. (Modified puncture [Title/Abstract]) OR (Paracentesis [MeSH]) OR (Biopsy, Needle [MeSH]) OR (Punctures [MeSH]).
3. 1 AND 2

### Inclusion and exclusion criteria

Inclusion criteria were as follows: randomized controlled trials (RCTs) involving children of any sex, age, or race, where the experimental group underwent a puncture improvement method and the control group received either an alternative or conventional puncture intervention. Studies published in Chinese or English were included. Eligible studies must have reported success rate as an outcome measure. Exclusion criteria were as follows: studies with insufficient or incomplete data; duplicate publications; and reviews or meeting minutes.

### Data extraction

All retrieved studies were imported into EndNote X9 software. Duplicate publications and those reusing data were excluded from this analysis. Two reviewers (HHM and CLH) independently screened the titles and abstracts to identify eligible studies. Full texts that met the inclusion criteria were further evaluated for final inclusion in the analysis. Any conflicts arising during the screening process were resolved through discussion or consultation with a third member (LLP) of the review team. Based on the characteristics of the included studies, we extracted the following key information from each randomized controlled trial (RCT): first author, year of publication, country, sample size, treatment and control interventions, and outcomes. The main outcomes indicators include Puncture success rate, Indwelling time, and Satisfaction rate.

### Quality of individual studies

The *Cochrane Manual of Systematic Evaluators 5.1.0* (13) was used as the criterion for literature quality evaluation, which included random allocation methods, hiding of allocation schemes, implementation of blind methods, integrity of outcome data, selective reporting of findings and other sources of bias. The judgements included “low risk of bias”, “unknown risk of bias”, and “high risk of bias”.

### Risk of publication bias

The risk of publication bias between studies was assessed using funnel plot symmetry and Egger regression tests.

### Statistics

In our meta-analysis, we rigorously assessed the heterogeneity between studies using *χ*^2^ test statistics. If *P* *>* *0.1* and *I*^2^ *<* *50%*, there was no statistical heterogeneity among the studies. A fixed effect model was used for meta-analysis. It indicated that there was heterogeneity among the different studies. The random effects model was selected, and the source of heterogeneity was further determined by subgroup analysis. If no source of heterogeneity could be found, descriptive analysis was used. Sensitivity analysis was used to evaluate the robustness of the results. Relative risk (RR) and 95% confidence intervals (CI) were used to estimate clinical efficacy, while continuous data were presented as standardized mean difference (SMD) and 95% confidence intervals. An inverted funnel diagram and Egger's test were used for publication bias analysis. A *p-value* less than 0.05 was deemed as significant. Statistical analysis was performed using Stata 16 software.

## Results

### Selected studies

**Figure 1:**
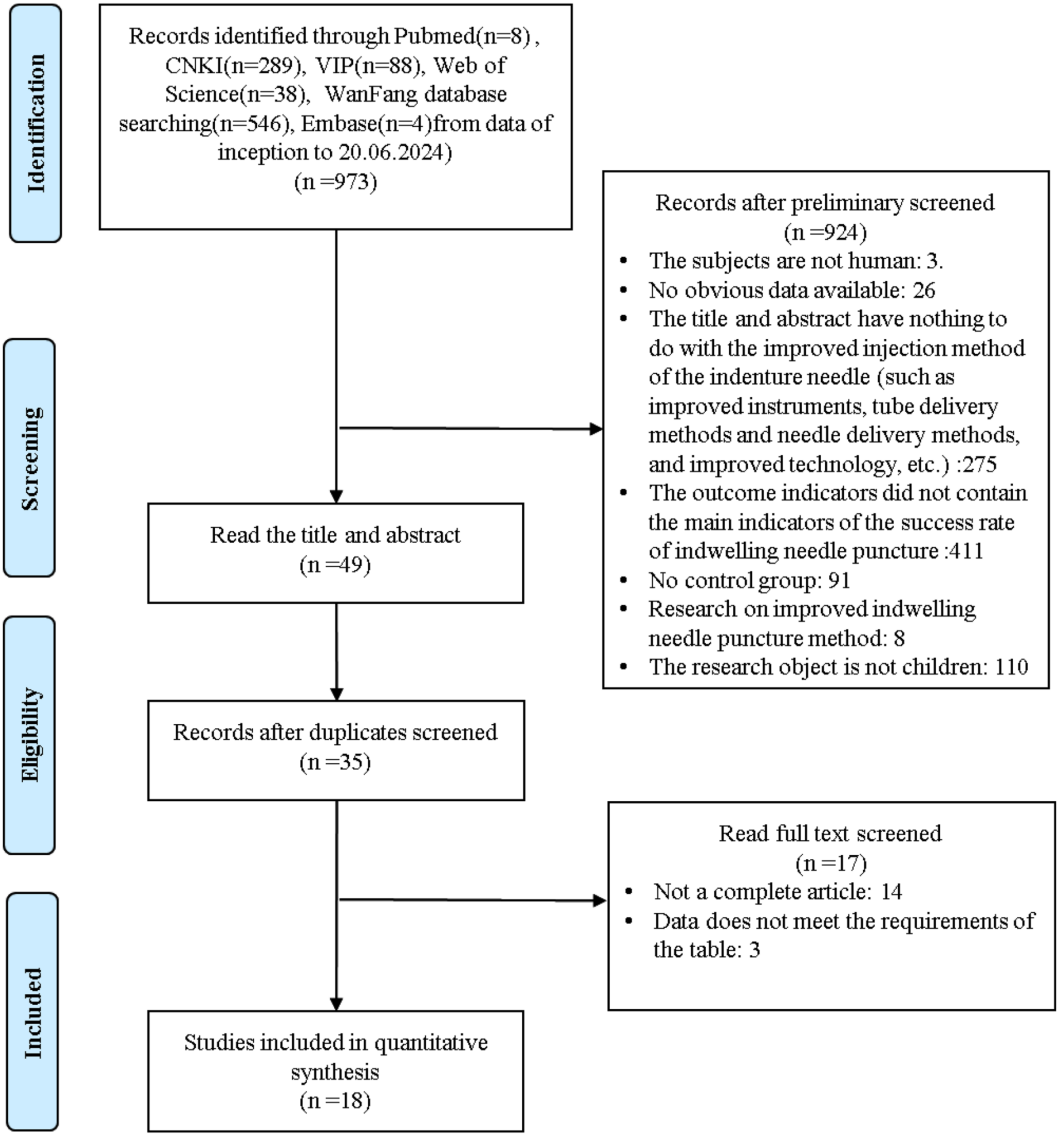
The literature screening process.

All studies identified from the search were exported to EndNote X9 Citation Manager, and we initially excluded 973 articles by title and abstract. Reasons for exclusion included: the subjects are not human(3); no obvious data available (26); the title and abstract have nothing to do with the improved injection method of the indenture needle, such as improved instruments, tube delivery methods and needle delivery methods, and improved technology, etc. (275); the outcome indicators did not contain the main indicators of the success rate of indwelling needle puncture (411); there was no control group (91); research is on the improved indwelling needle puncture method(8); the research object is not children(110). In addition, 14 duplicate articles were also deleted. After a full evaluation of the remaining articles, 17 further articles were excluded. Finally, we included 18 articles that passed the full-text review. Details of the selection process are shown in Figure 1.

### Study characteristics

The basic characteristics of the included literatures are shown in Table 1. All the 18 included studies were randomized controlled trials (RCTs), and all were from China. The inclusion and exclusion criteria and the evaluation criteria for each indicator are detailed in Supplementary Table S1.

**Table 1:** Demographic of all included studies.

First author	Year	Subjects of the study	Grouping method	*N*	Age		Sex(male)		Traditional group	Improved group	Improved type	Scalp or not
					Improved group	Traditional group	Improved group	Traditional group				
Li Jiangan	2010	Sick children	RCT	200	1 day–18 months	/	/	100	100	1	Yes	
Li Siqi	2021	Sick children	RCT	60	/	/	20	11	30	30	3	Not clear
Lv Qiongxiang	2009	Sick children	RCT	128	2 months–4 years	/	/	60	68	1	Yes	
Tao Xiaozhi	2009	Sick children	RCT	218	22 days–2.5 years	24 days–3 years	66	68	108	110	2	Yes
Wu Xiaomei	2019	Sick children	RCT	1,124	(2.87 ± 0.56)months	640	562	562	1	Not clear		
Zhuang Guiying	2011	Premature	RCT	120	/	/	/	/	60	60	3	No
Yuan Guifang	2011	Sick children	RCT	300	7.5 (2–15) months	160	150	150	3	Yes		
Dong Haiyan	2022	Sick children	RCT	380	1.62 ± 0.41 years	1.67 ± 0.43 years	93	94	190	190	3	Yes
Xue-yu Zhu	2013	Sick children	RCT	120	7.95 ± 2.51 years	7.84 ± 2.48 years	36	32	60	60	3	No
Jin-jin Wang	2015	Sick children	RCT	260	/	/	155	130	130	3	Yes	
Yu Zhenyan	2017	Sick children	RCT	150	2.3 ± 0.7 years	2.1 ± 0.5 years	/	/	75	75	2	No
AoChunMin	2013	Sick children	RCT	560	/	/	320	280	280	3	Not clear	
Xiu-ling Chen	2019	Sick children	RCT	50	2.42 ± 0.48 years	2.44 ± 0.49 years	13	14	25	25	3	No
Luo Jin	2013	Sick children	RCT	435	1 day–3 years 6 months	240	200	235	2	No		
Wang Jin	2016	Sick children	RCT	220	2 months–6 years	120	110	110	2	No		
Zhang Weihong	2019	Sick children	RCT	100	1.4 ± 0.8 years	1.6 ± 0.8 years	26	25	50	50	1	No
Liu Yan	2018	Sick children	RCT	96	1.97 ± 0.34 years	2.01 ± 0.41 years	29	30	48	48	3	No
Chen Xin	2014	Sick children	RCT	200	30 min–30 days	160	100	100	2	Not clear		

RCT, randomized controlled trial.

**Table 2:** RCT quality assessment (cochrane analysis bias assessment).

Author	year	Baseline comparability	Random allocation method	Allocation scheme hiding	Blind method	Integrity of the resulting data	Selectively report research findings	Other sources of bias
Li Jiangan	2010	Low bias	Low bias	Not clear	Not clear	Low bias	Low bias	Low bias
Li Siqi	2021	Low bias	Low bias	Not clear	Not clear	Low bias	Low bias	Low bias
Lv Qiongxiang	2009	Low bias	Low bias	Not clear	Not clear	Low bias	Low bias	Low bias
Tao Xiaozhi	2009	Low bias	Low bias	Not clear	Not clear	Low bias	Low bias	Low bias
Wu Xiaomei	2019	Low bias	Low bias	Not clear	Not clear	Low bias	Low bias	Low bias
Zhuang guiying	2011	Low bias	Low bias	Not clear	Not clear	Low bias	Low bias	Low bias
Yuan Guifang	2011	Low bias	Low bias	Not clear	Not clear	Low bias	Low bias	Low bias
Dong Haiyan	2022	Low bias	Low bias	Not clear	Not clear	Low bias	Low bias	Low bias
Xue-yu zhu	2013	Low bias	Low bias	Not clear	Not clear	Low bias	Low bias	Low bias
Jin-jin wang	2015	Low bias	Low bias	Not clear	Not clear	Low bias	Low bias	Low bias
Yu Zhenyan	2017	Low bias	Low bias	Not clear	Not clear	Low bias	Low bias	Low bias
AoChunMin	2013	Low bias	Low bias	Not clear	Not clear	Low bias	Low bias	Low bias
Xiu-ling Chen	2019	Low bias	Low bias	Not clear	Not clear	Low bias	Low bias	Low bias
Luo Jin	2013	Low bias	Low bias	Not clear	Not clear	Low bias	Low bias	Low bias
Wang Jin	2016	Low bias	Low bias	Not clear	Not clear	Low bias	Low bias	Low bias
Zhang Weihong	2019	Low bias	Low bias	Not clear	Not clear	Low bias	Low bias	Low bias
Liu Yan	2018	Low bias	Low bias	Not clear	Not clear	Low bias	Low bias	Low bias
Chen Xin	2014	Low bias	Low bias	Not clear	Not clear	Low bias	Low bias	Low bias

### Quality assessment

The quality assessment of this study is shown in Table 2.

### Results of meta-analysis Index

#### Puncture success rate

**Figure 2:**
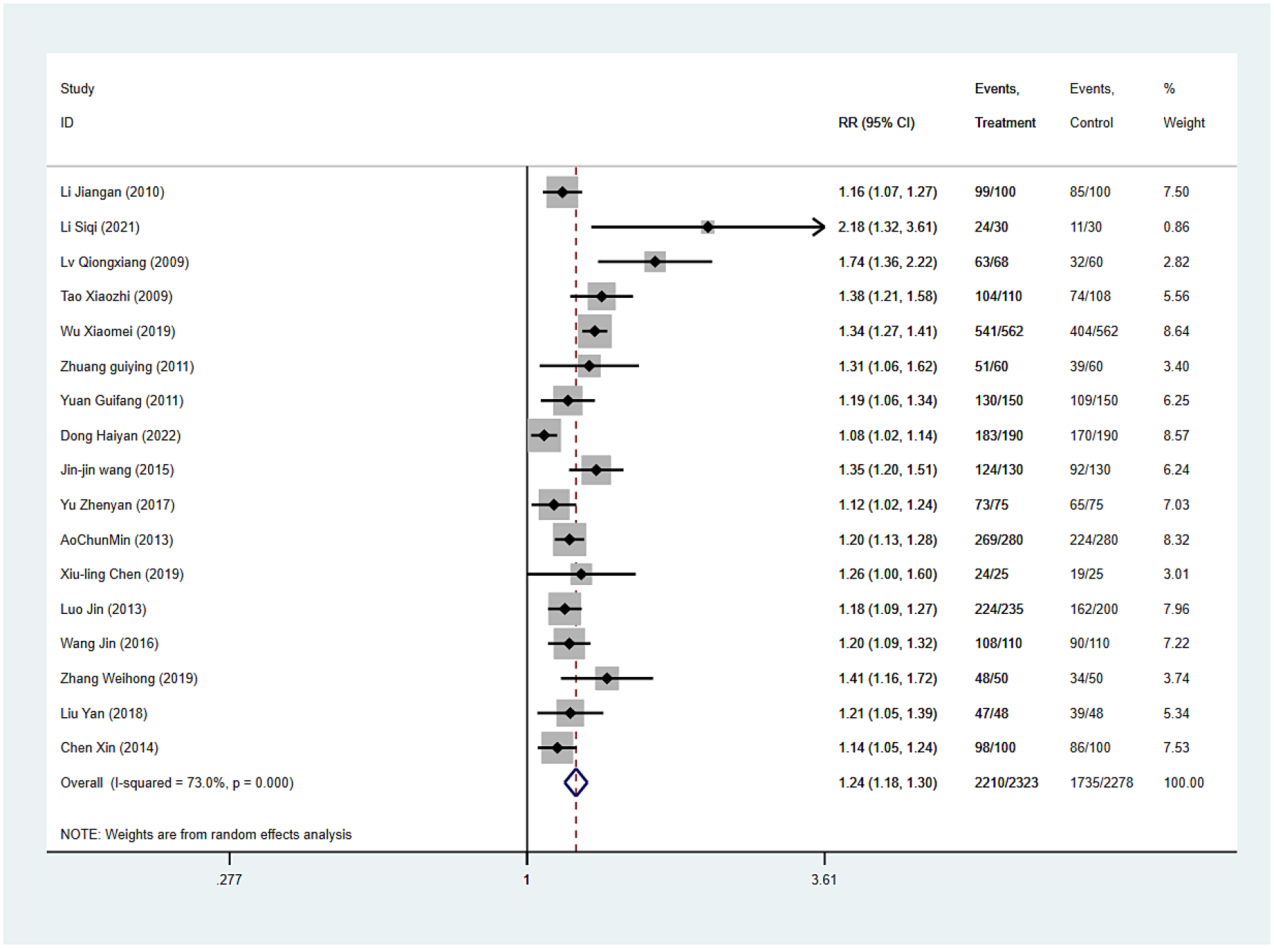
Puncture success rate forest map.

Puncture success rate was reported in all 17 studies, and the results of an inter-study heterogeneity test were *P* *<* *0.001* and *I*^2^ *=* *73.0%*. The meta-analysis using the random effects model showed that there was a statistically significant difference in the effective rate between the two groups [*RR* *=* *1.24 (1.18, 1.30), P* *<* *0.001*], and puncture success rate of the experimental group was *1.24* times that of the control group (Figure 2 and Table 3).

**Table 3:** Effect size.

Index	Heterogeneity test		Pooled RR(95%CI)/SMD(95%CI)	*P*
	*P*	*I* ^2^		
Puncture success rate	<0.01	73.00%	1.24 (1.18, 1.30)	<0.01
Indwelling time	<0.01	98.60%	3.65 (1.01, 6.30)	<0.01
Satisfaction rate	<0.01	70.80%	1.20 (1.10,1.31)	<0.01

#### Indwelling time

**Figure 3:**
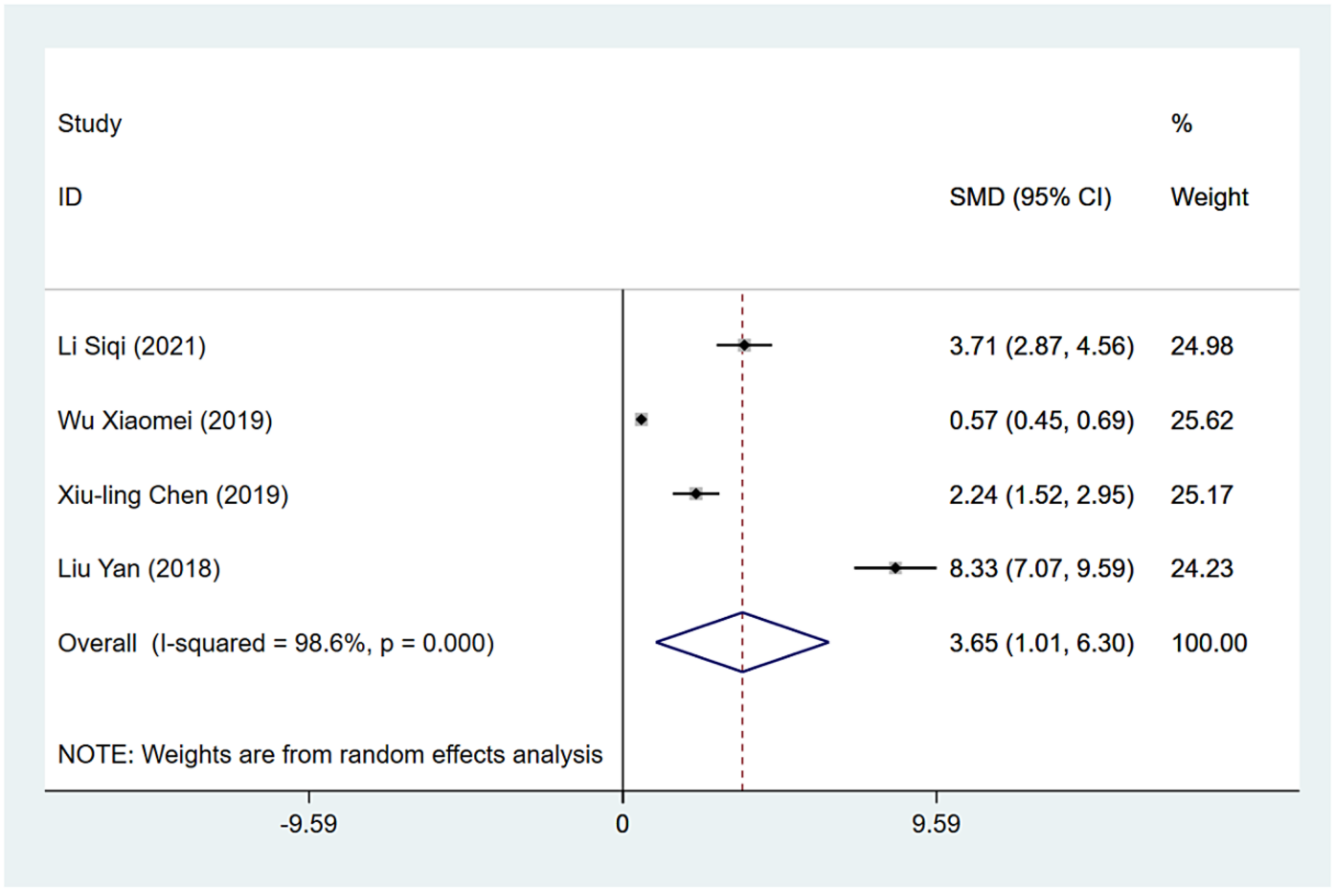
Indwelling time forest map.

Indwelling time was reported in four studies, and the results of the inter-study heterogeneity test were *P* *<* *0.001, I*^2^ *=* *98.6%*. A meta-analysis using random effects model showed that the indwelling time difference between the two groups was statistically significant [*SMD* *=* *3.65 (1.01, 6.30), P* *=* *0.007*], and the indwelling time of the experimental group was longer than that of the control group (Figure 3 and Table 3).

#### Satisfaction rate

**Figure 4:**
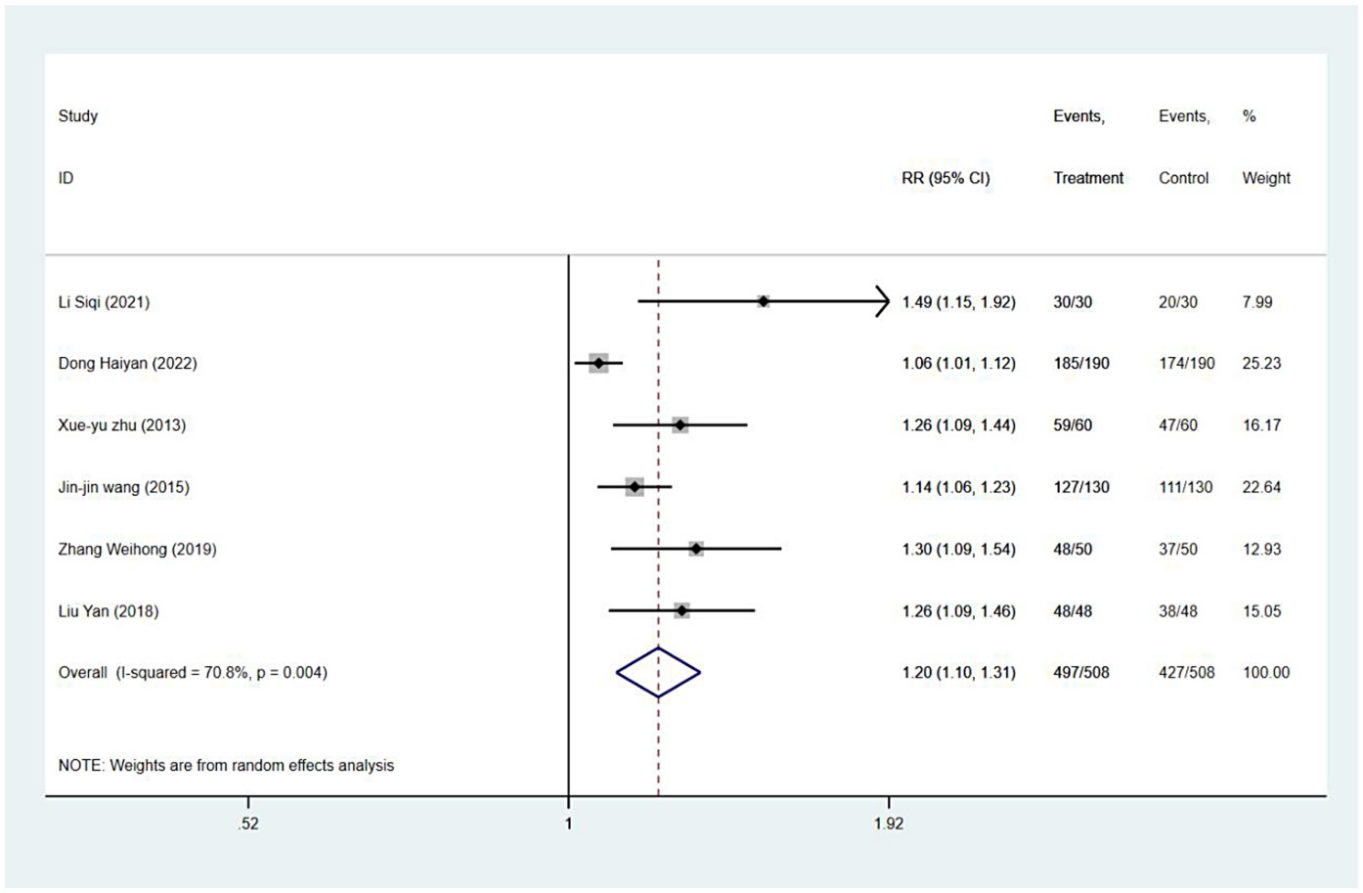
Satisfaction rate forest map.

Satisfaction rates were reported in six studies, and the results of the inter-study heterogeneity test were *P* *=* *0.004* and *I*^2^ *=* *70.8%*. A meta-analysis using the random effects model showed that there was statistically significant difference in the satisfaction rate between the two groups [*RR* *=* *1.20 (1.10,1.31), P < 0.001*] (Figure 4 and Table 3).

### Subgroup analysis

Regarding puncture success rate, satisfaction rate, the results of the subgroup analysis showed that improved type and scalp or not were the sources of heterogeneity. Puncture success rate of modified fixation was significantly higher than that of the control group [*RR* *=* *1.35 (1.19,1.52), P* *<* *0.01*]. The puncture site that included the scalp had a significantly higher success rate than the control group [*RR* *=* *1.26 (1.14,1.40), P* *<* *0.01*]. The puncture site that did not include the scalp had a significantly higher satisfaction rate than the control group [*RR* *=* *1.26 (1.61,1.38), P* *<* *0.01*] (Table 4).

**Table 4:** Summary of subgroup analysis results.

Index	Subgroup		Included studies	Heterogeneity test		RR (95%CI)	*P*
				*P*	*I* ^2^		
Puncture success rate	Improved type	1	4	0.01	77.90%	1.35 (1.19,1.52)	<0.01
		2	5	0.00	42.30%	1.19 (1.12,1.25)	<0.01
		3	8	0.01	68.70%	1.22 (1.13,1.32)	<0.01
	Scalp or not	Yes	6	0.01	83.20%	1.26 (1.14,1.40)	<0.01
		No	7	<0.01	0.00%	1.19 (1.14,1.25)	<0.01
		Not clear	4	0.01	82.50%	1.26 (1.13,1.40)	<0.01
Satisfaction rate	Improved type	1	1	/	/	1.30 (1.09,1.54)	<0.01
		2	0	/	/	/	/
		3	5	<0.01	72.20%	1.19 (1.08,1.30)	<0.01
	Scalp or not	Yes	2	<0.01	60.50%	1.10 (1.02,1.18)	0.694
		No	3	<0.01	0.00%	1.26 (1.16,1.38)	0.033
		Not clear	1	/	/	1.49 (1.15,1.92)	<0.01

Improved type: (1) **Modified fixation**: use sterile cotton ball pad indwelling needle, elastic cap fixed; (2) **Liquid connection indwelling needle**: saline was connected to the indwelling needle, injected before removing the needle core and sending the cannula, or injected into the child with shock and dehydration; (3) Improved method of removing core and feeding casing: improved position of finger holding indwelling needle, injection and withdrawal of needle core and delivery of sleeve finger.

### Publication bias

**Figure 5:**
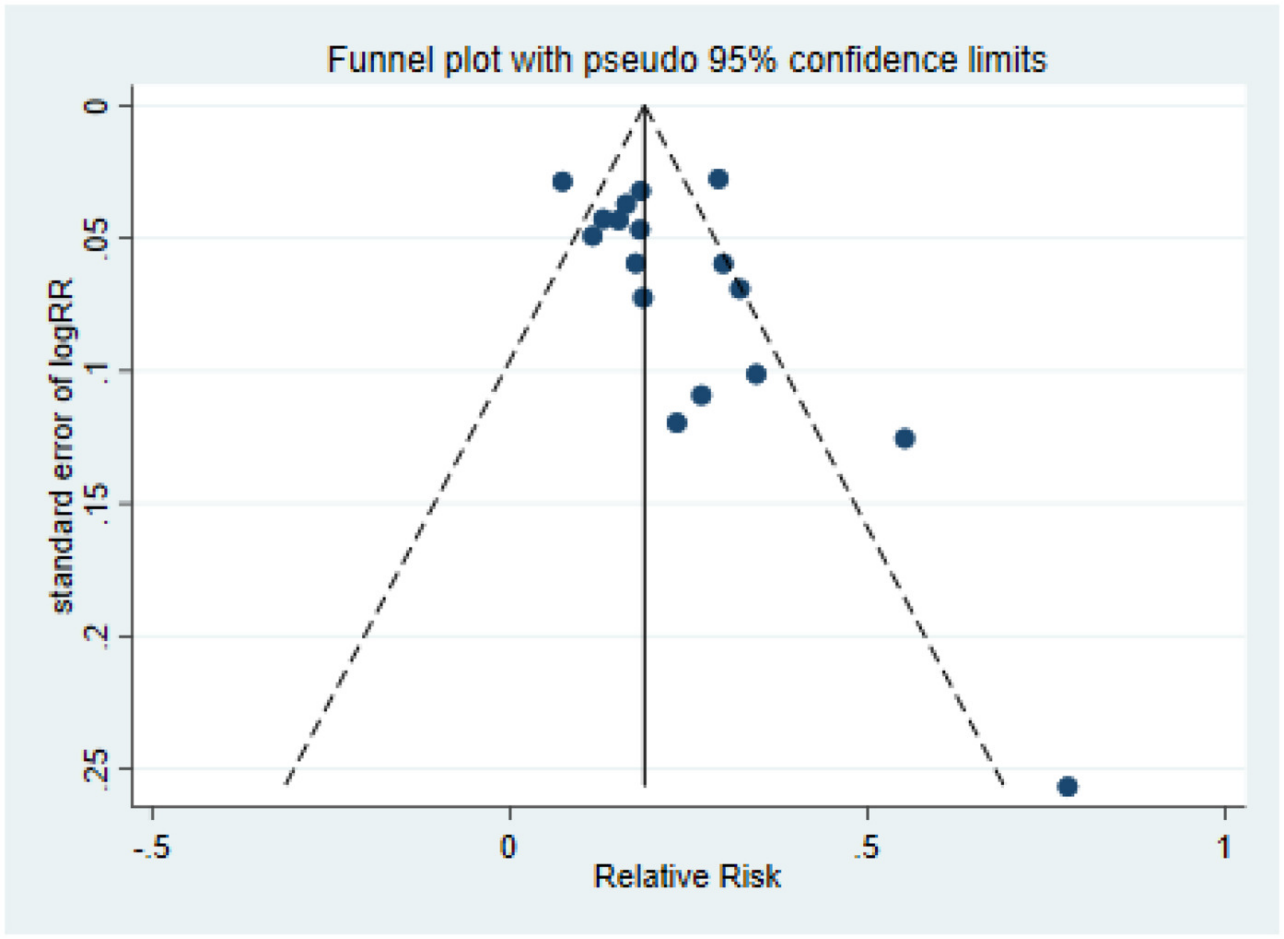
Funnel plot of puncture success rate.

The Egger test results for puncture success rate were *t* *=* *1.98, p* *=* *0.06*, and the scatter distribution of each study was basically symmetrical, indicating that there was no publication bias (Figure 5), the Rosental safe n number was 1,133.90. According to the common judgment criteria, the Rosenthal safety *N* value must be greater than (5k + 10) (where k is the number of included studies), and it was showed that the impact of publication bias on the results of this meta-analysis may be relatively small, and the research results are relatively stable and reliable. As fewer studies were included for other indicators, no evaluation of publication bias was performed.

### Sensitivity analysis

After deleting all studies one by one, the obtained 95% CI did not cross the invalid line, and the point of the combined effect size was still within the original 95% CI, indicating that the results of this study were relatively robust (Table 5).

**Table 5:** The puncture success rate of sensitivity analysis results.

Study omitted	Estimate	95% CI	
Li Jiangan (2010)	1.25	1.18	1.32
Li Siqi (2021)	1.23	1.17	1.29
Lv Qiongxiang (2009)	1.22	1.16	1.28
Tao Xiaozhi (2009)	1.23	1.17	1.30
Wu Xiaomei (2019)	1.23	1.16	1.29
Zhuang guiying (2011)	1.24	1.17	1.30
Yuan Guifang (2011)	1.24	1.18	1.31
Dong Haiyan (2022)	1.25	1.19	1.31
Jin-jin wang (2015)	1.23	1.17	1.30
Yu Zhenyan (2017)	1.25	1.18	1.32
AoChunMin (2013)	1.24	1.17	1.32
Xiu-ling Chen (2019)	1.24	1.17	1.31
Luo Jin (2013)	1.25	1.18	1.32
Wang Jin (2016)	1.24	1.17	1.31
Zhang Weihong (2019)	1.23	1.17	1.30
Liu Yan (2018)	1.24	1.17	1.31
Chen Xin (2014)	1.25	1.18	1.32
Combined	1.24	1.18	1.30

## Discussion

With the development of medical technology and the increase of clinical demand, the application scope of the indwelling needle is constantly expanding, After years of improvement, the materials and technology are more advanced and reliable, such as the new portable indwelling needle, the safe indwelling needle and the blood sampling indwelling needle. To summarize this study, the modified method of indwelling needle insertion technology on children mainly includes three methods: modified fixation; liquid connection indwelling needle; improved method of removing core and feeding casing. The results demonstrated that the modified indwelling needle techniques significantly improved puncture success rates, indwelling duration, and parental satisfaction. These benefits align with previous studies highlighting the advantages of technical optimizations in pediatric intravenous therapy.

1. The success rate of puncture has significantly increased. The results of this study showed that in terms of puncture success rate, the modified indwelling needle group was 1.24 times more effective that of the control group, indicating that the improvement in indentation needle puncture technology was conducive to the success of one-time puncture, improved patient satisfaction and extended number of retention days. Again, the advantages of improving the indentation needle are shown. Such as the improvement of indwelling needle fixation. The incidence of indwelling needle pressure ulcers can be reduced by a variety of methods, such as improving the fixed dressing, using sterile cotton, and using an elastic net cap (14), reducing blood return pollution (15), bringing down the incidence of slippage and dew at the junction (16) and improving the safety and patient comfort. Modified fixation can effectively reduce adverse reactions and complications, and prolong retention time, which is worth in clinical promotion (17, 18). The improved indwelling needle fixation technology can be carried out in the following ways: (1) Use specific materials and techniques to enhance the fixation effect and simplify the removal process; (2)Adopt a fixation method with intrinsic mechanical advantage to reduce the local pressure of the surrounding tissues and improve stability; (3)Use modern technology (such as 3D printing) to achieve high-precision and customized fixed solutions to improve overall stability and patient comfort (19). The second is liquid connection indwelling needles. Normal saline is added to the catheter, and normal saline is injected to fill the blood vessels (9); this helps to pull out the needle core, pushing it into the jacket tube; it also improves the success rate of puncture and patient satisfaction, and reduces patient pain. In addition, pay attention to heparin—for patients with liver insufficiency or heparin allergy, a saline filling catheter is recommended (20). The third method is the improved technique of removing the core and feeding casing. This can effectively improve the success rate of puncture by improving the needle core and casing, such as operating the needle return core sleeve with a single hand, improving the needle-holding and delivery technique, considering the specification and type of the indwelling needle, and further improving the tube insertion method. In order to improve the success rate of puncture, especially the indwelling needle core and casing link, the following aspects should be considered: (1)adopt the needle with a protection mechanism: although the needle with a protection mechanism (such as Insyte AutoGuard and Protective Acuvance) in venipuncture success rate is similar to the traditional needle, in the arterial puncture the back chamber is short, which leads to the blood reflux speed being too slow, which makes the insertion process more difficult. Therefore, when selecting the needle head, different types of puncture requirements should be considered, and needles suitable for specific use should be selected (21). (2) Newly designed catheter use: The pre-bent catheter (UPC) has a better fixation and lower infection rate than the conventional straight neck catheter (USC). This suggests that using the newly designed catheter can improve on the complications after puncture, thus indirectly improving the puncture's success rate (22). (3) Continuous training and standardized teaching: according to the research, continuous training and standardized teaching for new nurses are an effective means to improve venipuncture skills (23). This shows that through systematic training and practice, the puncture skills of medical staff can be effectively improved thus improving the success rate of puncture (24).
2. The retention time was effectively prolonged 3.65 (1.01, 6.30) days. This can primarily be attributed to the impact of various enhanced techniques on the stability of indwelling needles and vascular protection. The improved fixation method mitigates complications arising from indwelling needle displacement and compression by distributing localized pressure and enhancing fixation stability, thereby extending the duration of indwelling time (14–16). The liquid connection indwelling needle technique pre-fills the catheter with normal saline to ensure vascular patency and facilitate the withdrawal of the needle core and advancement of the cannula. This approach minimizes vascular damage, maintains the patency of the indwelling needle, and consequently prolongs its indwelling time. From a clinical perspective, extended indwelling time reduces the frequency of indwelling needle replacements, alleviating patient discomfort from repeated punctures while decreasing the workload for medical staff. Particularly for patients undergoing long-term infusion therapy, this enhances the continuity and efficacy of treatment. However, due to the extremely high data heterogeneity (*I*² = 98.6%), these findings should be interpreted with caution. Given the limited number of included datasets, subgroup analysis was not feasible. This heterogeneity may stem from variations in nursing standards and levels across institutions, as well as inconsistencies in observation times and evaluation criteria for indwelling duration across studies.
3. Patient satisfaction has demonstrably enhanced. The rise in the success rate of punctures and the extended duration of indwelling time have effectively minimized patient discomfort and inconvenience. Additionally, the refinement of techniques during procedures, including more ergonomic fixation methods and diminished puncture-related pain, have substantially elevated the overall treatment experience for patients.
4. There have been other developments regarding the improvement of the indwelling needle. For example, a precision indwelling needle with dual anticoagulant/haemostasis function was developed by anchoring the anticoagulant heparin coating and the haemostatic hydrogel coating to the inner and outer surface, respectively, on the external surface of the indwelling needle, to achieve implantation patency and prevent uncontrolled bleeding caused by needle extraction (25). In addition, the psychological care of children can improve the success rate of puncture and reduce the risk of complications (26). The use of communication skills and child-friendly language and actively communicating with children create a sense of trust, helping children relax emotionally, divert their attention, and improve the degree of cooperation (27).

To sum, the improved indwelling needle technique helps to improve the success rate of disposable puncture, prolongs the indwelling time and improves parental satisfaction. These improvements can not only improve the patient's treatment experience, but also improve the overall efficiency and effectiveness of the healthcare system (28). Through the modified fixation, the liquid connection indwelling needle, and the improved method of removing core and feeding casing, indwelling needle insertion technology is improved to form a more advanced and effective new mode of intravenous infusion in children.

## Study limitation

All included studies were from China due to the inclusion and exclusion criteria, which may cause selection bias in this study.

## Conclusion

Our results show that the improved indwelling needle technique facilitates the benefit of improving the disposable puncture success rate of the needle, extending the indwelling time and improving parental satisfaction.

## Data Availability

The original contributions presented in the study are included in the article/Supplementary Material, further inquiries can be directed to the corresponding author.
