# Supplementary material for: Effect of modified indwelling needle insertion techniques in Chinese children: a systematic review and meta-analysis
Source: Front Pediatr. 2025 Jul 9;13:1579303. doi: 10.3389/fped.2025.1579303 (PMC12283645; doi:10.3389/fped.2025.1579303)
Supplement: Supplementary file 1 [file Table1.docx]

| Supplementary Table 1 Baseline characteristics of included studies | | | | | | | |  |
| --- | --- | --- | --- | --- | --- | --- | --- | --- |
| First author | Year | reigon | Inclusion criteria | Exclusion criteria | Indwelling needle model | Observe the metrics | Specific methods of puncture | |
|  |  |  |  |  |  |  | Traditional Group | Improved Group |
| Li Jiangan | 2010 | China | NA | NA | 24Gd | 1.Puncture success rate, 2.Indwelling time | Others assisted in the fixation of the child's head, 5 to 10 needle 1 mm, the left hand to send the jacket into the blood vessel, the right hand with the wing of the needle core, apply fixed, paper tape around the head | 5 to 10 injection 1 mm, the left hand holds the head of the child with the left hand, the right hand holds the needle wing into the blood vessel and the needle core, the dressing is fixed, the elastic mesh cap is fixed |
| Li Siqi | 2021 | China | NA | NA | NA | 1.Puncture success rate, 2.Indwelling time, 3.Parental satisfaction | 15 to 30 needle 1-2mm, one hand fixed the needle core for support, the other hand sent all the casing into the vein, return the needle core, do fixation | The left hand fixed the skin, the right hand punctured into the blood vessel on the original basis of 15 to 30, push 2mm forward, the needle core back, the left hand tightened the skin to keep the blood vessel and the needle entry point, then sent to the needle core, the blood back exits the needle core, and the dressing is fixed |
| Lv Qiongxiang | 2009 | China | Children admitted to our hospital who require intravenous fluid therapy | NA | 24 | 1.Puncture success rate, 2.Occurrence of adverse reactions | Others assisted in the fixation of the child's head, 15 to 20 needle 2-3 mm, the left hand fixed the outer tube, the right hand off the needle core and then send the outer tube into the blood vessels, tape fixed | One person fixed the head of the child with one hand and tighten the skin, make the blood vessels into a straight line, the other person into the needle 2-3mm, the right hand back the needle and the left hand to send the outer tube into the blood vessels, the puncture point for the central dressing fixed, tape wound, bandage embedded |
| Tao Xiaozhi | 2009 | China | From January 2004 ~ December 2005, 108 children requiring intravenous treatment were set as the traditional group, and the traditional puncture method of intravenous indwelling needles was used, and 110 children requiring intravenous treatment from January 2006 ~ December 2007 were set as the improved group | NA | 22GA /24GAY | 1.Puncture success rate, | Right hand needle 60 into the needle, after the blood, then into a little needle. The left thumb and index finger hold the cannula into the blood vessel, and at the same time, the right thumb and index finger withdraw the needle core. When there is blood back, fix the wing of the needle, remove the needle core, and fixed with transparent paste. | The injection is injected into the left thumb and index finger of the cannula into the blood vessel, and the right thumb and index finger of the cannula is sent into the vascular syringe for blood return, and then the needle core is pulled out and fixed with transparent tape and tape |
| Wu Xiaomei | 2019 | China | Infants aged < 6 months who were hospitalized in the pediatric department of the Second People's Hospital of Pingliang City from January 2011 to April 2013 and required VIN infusion therapy were selected | NA | 24Y | 1.Puncture success rate, 2.Indwelling time, 3.Occurrence of adverse reactions | Routine VIN puncture method, the puncture point is covered with the central dressing and fixed with tape | Innovative venipuncture method, improved fixation method to fix the needle handle with non-woven tape, with sterile cotton ball under the handle; the second tape cross wound fixed the needle handle, pay attention to avoid the needle eye |
| Zhuang guiying | 2011 | China | From March 2008 to November 2009, 120 premature infants were admitted to the hospital from 30 to 15 days after delivery | NA | 24G | 1.Puncture success rate | The needle raised the skin, see the blood parallel injection needle 1 ~2 mm, the left hand fixed the needle, the right hand removed the needle core about 5 mm, and send the jacket tube into the blood vessel along the direction of the blood vessel. Press the left thumb under the needle stem, exit the right hand from the needle core, and apply the fixed needle | The axillary vein back 0.5~1.0 cm, the needle stabbed into the lower skin, lower the tail of the needle, the needle body parallel to the vein, see blood return to slide l ~2 mm, the right thumb tip against the Y tube, index finger hook the needle core 0.5 ~1.0cm, the thumb forward push the outer tube along the vascular direction into the blood vessel, pull out the needle core, apply the fixed needle |
| Yuan Guifang | 2011 | China | From January 2008 ~ June 2009, 300 cases of superficial temporal vein puncture were performed in hospitalized children in our department | NA | 24G | 1.Puncture success rate | Others assisted to fix the head of the child, tighten the skin at the front of the puncture point in the left hand, stabbed the needle 20 ~30 in the right hand, and then lowered the Angle less than 15 to directly puncture the blood vessels. Transparent application and fixation | Assist to fix the head of the child, hold the indwelling needle in the right hand, the needle tip inclined plane upward, and the skin into 20-30 into the skin, and then reduce the Angle less than 15 into the blood vessel, push forward 1-2mm, the right hand fixed the needle handle, the left hand sent the cannula into the vein, and pull out the needle core. Transparent application and fixation |
| Dong Haiyan | 2022 | China | (1) Meet the indications for venipuncture; (2) The skin at the puncture site is intact; (3) All family members have informed consent. | (1) Low family cooperation; (2) children with serious diseases; (3) Patients with other organic diseases | 24G | 1.Puncture success rate, 2.Indwelling time, 3.Parental satisfaction | The left hand is fixed, the right hand holds the needle at the Angle of 1 5 ~30, reduce the needle Angle, and then enter the needle 1 ~2 mm along the vascular direction, the left hand sends the tube, the right hand exits the needle core, transparent application is fixed | The left index finger and thumb are in the shape of "C", fix the skin of the puncture site of the child in the opposite direction, enter the needle at 1 5 -30, reduce the injection Angle and enter the needle by 1 ~2 mm along the vascular direction; push the needle wing in the right hand, while the thumb and index finger against the Y hose intersection, and push the hose along the vascular direction; the transparent dressing is fixed |
| Xue-yu zhu | 2013 | China | NA | NA | 26 | 1.Puncture success rate, 2.Parental satisfaction | In the control group, the needle tip was upward at 20 angles to the skin and did not change the angle after injection. See the blood back all the coat into the blood vessel, the left hand fixed needle handle, the right hand pull out the needle core, apply bright film fixed | The left hand tighten the skin, the right hand holds the needle and the skin into 45 Angle fast injection, reduce the Angle to 20 along the direction of the blood vessel. See the blood back to all the coat into the blood vessels, the left hand fixed needle handle, the right hand pull out the needle core, apply bright film fixed. |
| Jin-jin wang | 2015 | China | Children with clear consciousness, good spirits, free movement, and stable vital signs, | Critically ill and seriously ill children are excluded | 24 | 1.Puncture success rate, 2.Parental satisfaction | The left hand tightened the skin, the right thumb and middle finger pinch the tail of the needle core, the needle tip oblique upward puncture, and then into the needle 2 mm, with the right hand into the vein, the transparent dressing step fixed, the right hand removed the needle core | The left hand tightened the skin, the right thumb against the tail of the needle core, the middle finger holding the right flank into the blood, the puncture Angle is slightly reduced, the needle tip slightly lifted, then the needle 2 mm, put the needle tube, the forward cannula all into the vein, the transparent dressing is fixed, the right hand withdrew the needle core |
| Yu Zhenyan | 2017 | China | During the same period, the pediatric outpatient clinic was assessed as moderately dehydrated | NA | 24G | 1.Puncture success rate, | The left hand is fixed and tightened the skin, the right hand 15 ~ 30 Angle injection, reduce the Angle, the needle 1 ~ 2 mm, a little needle, the right thumb and middle finger fixed the needle core, the index finger will be the outer tube into the vein | After the normal saline was filled with the indwelling needle, the puncture was performed. After the blood return, the indwelling needle continued to enter the needle along the blood vessel 1 ~ 2 mm, the indwelling needle core was withdrawn a little, the thumb and middle finger of the right hand were fixed, and the outer tube was sent into the vein with the index finger |
| AoChunMin | 2013 | China | NA | NA | 24GY | 1.Puncture success rate, | The right hand holds the needle 15~30 angle needle, see the blood, and then a little needle, with the two hands to send the outer tube into the vein, need to be fixed by others | The left hand tense the child's skin to fix the blood vessels, the right hand uses the front and rear holding needle, 30 to 40 Angle straight stab the blood vessel, lower the needle tail into the needle, see the blood and the blood vessel parallel forward 2-3mm, the right thumb and index finger hold the y tube ready to move forward, at the same time slide back the needle handle, the tape fixed |
| Xiu-ling Chen | 2019 | China | NA | NA | NA | 1.Puncture success rate, 2.Indwelling time, 3.Parental satisfaction | Conventional intravenous needle puncture method, after the puncture | The left hand tightened the skin to fix and straighten the blood vessels, the right hand 35 to 45 needle, after the needle Angle down 5 to 10, the distance is about 2 mm. Fixed the coat handle with the left hand, the tip of the right hand into the tube sleeve, the handle of the coat continues to move forward to about 1mm from the root according to the 5 Angle, push the coat handle to the left with the right hand, slowly pull out the needle core, and fix it with a disposable film |
| Luo Jin | 2013 | China | NA | NA | 24G | 1.Puncture success rate, | 15-30 Angle injection, after the blood return, the one hand fixed the needle seat, the other hand back the needle core 3-3.5mm, and then all the hose into the blood vessels. Remove the pin core and fix it properly | 2 people operated, 5-10 angle injection, after seeing the blood, exit the needle core 3-3.5mm, and then send 1-2mm forward to ensure that the cannula entered the blood vessel, injected 0.9% NaCl injection, at the same time, all the operator tube into the blood vessel, remove the needle core, and be fixed |
| Wang Jin | 2016 | China | NA | NA | 24G | 1.Puncture success rate, | 15-30 Angle injection, after the blood return, the one hand fixed the needle seat, the other hand back the needle core 3-3.5mm, and then all the hose into the blood vessels. Remove the pin core and fix it properly | 2 people operated, 5-10 angle injection, after seeing the blood, exit the needle core 3-3.5mm, and then send 1-2mm forward to ensure that the cannula entered the blood vessel, injected 0.9% NaCl injection, at the same time, all the operator tube into the blood vessel, remove the needle core, and be fixed |
| Zhang Weihong | 2019 | China | (1) All patients who require intravenous indwelling needle treatment after examination at our hospital and whose parents agree to receive treatment at our hospital. (2) The patient needs it during the treatment at our hospital Be accompanied by a parent. (3) The patient had no experience of venipuncture in other hospitals before treatment in this hospital. (4) After examination, the patient's intelligence is normal. | (1) The patient's illness is mild, and there is none Serious diseases, such as medical diseases, occur (2) Home Long informed but not consented to participate in this study. 50 patients in the study group In the middle, there were 26 males and 24 females, aged 0. 5 ~ 3 years old, average (1. 4 ±0. 8) years, the patient's condition: bronchitis 12 cases, respiratory tract sensation 20 cases of infection, 18 cases of gastroenteritis; Of the 50 patients in the control group, 25 were males Case, 25 females, age 0. 6 ~ 3 years old, average (1. 6 ± 0. 8) years old,Patients' symptoms: 18 cases of bronchitis, 14 cases of respiratory tract infection,Gastroenteritis in 18 cases. | NA | 1.Puncture success rate, 2.Parental satisfaction | Hold the needle 15 ~ 30 in the right hand, after seeing the blood, insert a little needle into the inside; finally, the intravenous needle is fixed with the right hand, send the outer tube into the venous vessel of the patient with the left hand, and fix the needle with tape | Fixed the left hand, and the corner of the right hand directly penetrates the blood vessel; after seeing the blood back, lower the tail of the indwelling needle, push the needle, hold the left hand, hold the Y tube to push forward, and the middle finger slides back from the needle handle; transparent application for fixation |
| Liu Yan | 2018 | China | (1) All children need infusion therapy or nutritional supply; (2) The parents of the children voluntarily accepted the study and signed the informed consent form | (1) Systemic skin damage or skin-related diseases; (2) children after ostomy surgery; (3) Pick-up Children receiving phototherapy | NA | 1.Puncture success rate, 2.Indwelling time, 3.Occurrence of adverse reactions | The traditional intravenous indwelling needle puncture technique puncture the child, and after the puncture is completed, the child is fixed with disposable film | Left hand skin fixed straightening, right hand 30 ~ 45 Angle needle puncture, appropriate after see blood needle Angle reduction, is 5 ~ 10 Angle needle left hand fixed coat handle, right hand back a little needle core, at the same time, the tip into the casing, coat handle according to about 5 continue to push forward to the root 0.1 cm right hand tilt left coat handle, left hand fixed processing, pull out the pillow core, fixed |
| Chen Xin | 2014 | China | This study collected 200 children admitted to the neonatology department from January 1, 2012 to December 30, 2012 | NA | 24G | 1.Puncture success rate, 2.Indwelling time. | 15 ~ 30 Angle into the needle, see the blood back, one hand fixed the needle seat, the other hand back the needle core, all the hose into the blood vessel. Remove the needle core and apply it to fix it transparently | After 2 ~ 3mm of injection, the blood was returned from the syringe, slowly sending the tube and backing the needle core, and the blood return was good again. 0.9%NS was injected to ensure that the needle core was removed after the casing was in the vessel. Clear apply stick fixed |
