# Supplementary material for: Effect of modified indwelling needle insertion techniques in Chinese children: a systematic review and meta-analysis
Source: Front Pediatr. 2025 Jul 9;13:1579303. doi: 10.3389/fped.2025.1579303 (PMC12283645; doi:10.3389/fped.2025.1579303)
Supplement: Supplementary file 2 [file Table2.docx]

| **Supplementary Table 2** |  |
| --- | --- |
| Database | Retrieval strategy |
| embase | (intravenous detaining needle or intravenous indwelling needle or venous indwelling needle or venous indwelling trocar or indwelling needle or PVIN).mp. [mp=title, abstract, heading word, drug trade name, original title, device manufacturer, drug manufacturer, device trade name, keyword heading word, floating subheading word, candidate term word] AND (Modified puncture or Paracentesis or Biopsy, Needle or Punctures).mp. [mp=title, abstract, heading word, drug trade name, original title, device manufacturer, drug manufacturer, device trade name, keyword heading word, floating subheading word, candidate term word] |
| web of sicience | ((ALL=(Modified puncture)) OR TS=(Paracentesis)) OR TS=(Biopsy, Needle)AND (((((ALL=(intravenous indwelling needle)) OR ALL=(intravenous detaining needle)) OR ALL=(venous indwelling needle)) OR ALL=(venous indwelling trocar)) OR ALL=(PVIN)) OR ALL=(indwelling needle) |
| CNKI | (Theme: indwelling needle + indwelling needle puncture + indwelling needle infusion) AND (Theme: modified + modified method + modified method) AND (Theme: puncture + venipuncture + percutaneous puncture + puncture site + puncture needle) |
| Wan Fang | (All =intravenous detaining needle OR all =intravenous indwelling needle OR all =venous indwelling needle OR. All =venous indwelling trocar OR all =venous indwelling cannula needle OR all = peripheral venous indwelling needle OR all =venous indwelling needle OR all = cannula needle OR all = indwelling needle) AND (all = modified puncture method |
| Wei Pu | [((((((((((Arbitrary field =intravenous detaining needle) OR arbitrary field =intravenous indwelling needle) OR arbitrary field =venous indwelling needle) OR arbitrary field =venous indwelling trocar OR arbitrary field =venous indwelling cannula needle OR arbitrary field = peripheral venous indwelling needle OR arbitrary field =venous cannula needle OR arbitrary field = cannula needle OR Any field = indwelling needle) AND Any field = Modified puncture method](https://vpnlib.cmc.edu.cn/http/77726476706e69737468656265737421fcfe43d224217e596e468aa395/Qikan/search/index?LngMySearHistoryIdGuid=28359c93-e6e5-4604-940f-831f30ffcb5f&from=Qikan_Article_History) |
